# The OPERA project: a harmonized multinational real-world ART database across Italy and Germany (2016–2020) and a descriptive snapshot of contemporary clinical practice

**DOI:** 10.3389/frph.2026.1822310

**Published:** 2026-04-30

**Authors:** Andrea Francavilla, Chiara Pavoni, Mariangela Fumarola, Eleonora Sfreddo, Francesco Maria Fusi, OPERA Working Group

**Affiliations:** 1 FROM - Research Foundation Bergamo Hospital - ETS, Bergamo, Italy; 2 Department of Maternal Fetal and Pediatric Medicine, ASST Papa Giovanni XXIII, Bergamo, Italy

**Keywords:** assisted reproductive technology, data harmonization, multicenter database, ovarian stimulation, real-world data

## Abstract

**Introduction:**

Assisted reproductive technology (ART) practice varies widely across centers and evolves rapidly, often outpacing the generation of randomized evidence. OPERA (Observational ProjEct for a Research database for ART procedures) was developed to create a harmonized, multinational real-world database derived from routine clinical ART documentation and to provide a structured overview of contemporary clinical practice.

**Materials and methods:**

OPERA is a retrospective, multicenter database including 18 IVF/ICSI centers (9 in Italy and 9 in Germany) using a shared electronic platform (Meditex). All second- and third-level ART cycles performed between January 1, 2016 and December 31, 2020 were eligible for inclusion. Data were extracted from Meditex, harmonized into four relational domains (Baseline, Stimulation, Laboratory, and Pregnancy outcomes), and anonymized prior to aggregation. After data cleaning (duplicate removal, exclusion of unlinked frozen embryo transfer cycles, and cycles with incomplete follow-up), 64,081 ovarian stimulation cycles and 31,027 intended frozen embryo transfer cycles were included. Analyses were descriptive, and no imputation was performed.

**Results:**

Median female age was 37 years (IQR 33–39), and median BMI was 22.7 kg/m². Antagonist protocols were used in 78.4% of cycles. Clinical pregnancy rates were 33.8% for fresh embryo transfers and 34.2% for frozen embryo transfers, with corresponding live birth rates of 25.8% and 24.3%.

**Discussion:**

OPERA provides a large, harmonized real-world ART dataset enabling a comprehensive overview of clinical practice across multiple centers. While the descriptive design does not allow causal inference, it supports benchmarking and hypothesis generation for future analytical studies, acknowledging inherent limitations related to variable completeness and privacy-preserving data structure.

## Introduction

Infertility is a common finding and determines a substantial individual and societal burden (1). Over the last decades, ART has become an essential part of infertility care, with increasing treatment volumes and evolving clinical and laboratory strategies. Yet ART is also one of the most practice-variable areas of medicine. Decisions regarding protocol selection, gonadotropin strategy, trigger approach, luteal support, laboratory workflow, embryo culture, and the balance between fresh transfer and freeze-all policies can differ across countries, centers, and even physicians within the same institution. Clinical practice also evolves rapidly, often faster than randomized evidence can accumulate and be translated into guidelines.

In this landscape, real-world data (RWD) can complement classical evidence generation (2). Routine clinical data can reveal how treatments are used in everyday practice, across broader and often more complex patient populations than those enrolled in trials. -Importantly, observational data can capture heterogeneity: the diversity of ovarian reserve, infertility etiologies, comorbidities, laboratory strategies, and center-level policies that shape outcomes in routine care.

National and regional ART registries provide indispensable surveillance and public reporting (3). However, registry systems are not always designed to capture the depth of clinical and laboratory detail needed for many research questions—particularly questions involving protocol nuance, embryology variables, treatment sequences, or cumulative outcomes derived from multiple transfers. Variable completeness may differ across reporting frameworks, and some clinically relevant concepts (e.g., adjuvant use, detailed embryo development counts, or specific reasons for cancellation) may be unavailable or inconsistently reported.

Despite the availability of national ART registries, access to harmonized, patient-level, multicenter real-world datasets remains limited. This represents an important gap in the literature, as such datasets are needed to support reproducible observational research and benchmarking of contemporary ART practice across centers and countries. To address these gaps, a real-world database designed to provide a harmonized and reproducible framework for the analysis of ART clinical practice across countries is of foremost importance. OPERA (Observational ProjEct for a Research database for ART procedures) was created in this space: a multicenter, multinational effort to assemble a harmonized dataset from routine electronic records, with predefined variables, explicit governance, and a repeatable extraction pipeline. OPERA is not intended to replace registries; rather, it is designed as an enabling platform for reproducible real-world research and for descriptive benchmarking of contemporary ART practice at a level of detail that is often difficult to achieve in surveillance databases (4).

The aim of this manuscript is twofold. First, we describe how OPERA was established—where the data come from, how they are processed, how privacy is preserved, and how the project is governed. Second, we provide a descriptive “photograph” of the database to illustrate what OPERA captures and what it can support, using cycles performed in participating centers between 2016 and 2020.

## Materials and methods

### Study design and overarching objectives

OPERA is a retrospective, observational database initiative, promoted by Fondazione per la Ricerca Ospedale di Bergamo (FROM)—ETS, created to generate RWD describing standard clinical practice in ART and to support a portfolio of non-interventional studies. This report was prepared in line with STROBE and RECORD recommendations for observational studies using routinely collected health data (5, 6). The project was designed from the outset around two guiding principles:

1. Harmonization at scale—a repeatable extraction pipeline anchored in a shared electronic platform used in routine care;
2. Governance and appropriate use—a formal Scientific Committee structure overseeing project priorities, study feasibility, analysis plans, and publication outputs.

The primary objective of OPERA is to build and maintain a large, harmonized ART database capable of characterizing patient baseline profiles, treatment pathways (including stimulation, laboratory activity, and embryo transfer), and outcomes, while also capturing selected health-economics descriptors when available. Secondary objectives include enabling comparative effectiveness and safety analyses of specific components of ART care (e.g., ovarian stimulation strategies and gonadotropins, endometrial preparation approaches, and embryological variables associated with pregnancy outcomes) in future analytical studies based on the OPERA dataset, as well as supporting methodological work in observational inference and data quality.

### Setting, participating centers, and observation window

OPERA includes 18 IVF/ICSI clinics, with the participation of 9 centers in Italy and 9 in Germany. Eligible records included all second- and third-level ART cycles performed in participating centers between January 1, 2016 and December 31, 2020 and documented in the shared electronic platform. Cycles were included if they contained sufficient information to be harmonized within the predefined OPERA data structure. For the descriptive snapshot presented here, duplicate records, FET cycles not linkable to an originating ovarian stimulation cycle, and cycles with incomplete follow-up were excluded.

### Source system and routine documentation

All participating centers routinely document their ART activity in Meditex, an electronic platform used for the management of any intervention on infertile couples, including each cycle tracking and documentation of baseline characteristics, ovarian stimulation and triggering, laboratory activity, embryo transfer procedures, and pregnancy-related outcomes. The use of a shared platform substantially reduces heterogeneity at the data source level, enabling standardized field selection and mapping across centers while still acknowledging that local workflows and minor platform customizations may influence completeness and coding patterns.

### Data extraction pipeline and relational data structure

#### Standardized extraction

Data were extracted using a standardized extraction model applied across participating centers, ensuring consistency in field selection and variable mapping. Extracted data were then organized into a relational structure reflecting the clinical pathway.

#### Relational tables

OPERA data are organized into four relational domains:

1. Baseline: demographics and baseline prognosis factors (e.g., age, BMI, infertility duration, infertility type and factors, ovarian reserve markers such as AMH; baseline hormones when available; smoking status; sperm source).
2. Stimulation: protocol category (e.g., antagonist or agonist variants), gonadotropin class, total dose, stimulation duration, adjuvant treatments, peak hormone values when available, trigger strategy, number of oocytes retrieved, OHSS-related indicators, cancellation status and reasons, luteal support.
3. Laboratory: insemination method (IVF, ICSI, or both), number of oocytes inseminated/injected, fertilization data (e.g., 2PN where available), embryo development results, cryopreservation flags and counts, PGT-A indicator when available, day and number of embryos transferred, and remaining cryopreserved embryos.
4. Pregnancy outcomes: biochemical pregnancy, clinical pregnancy, miscarriage/pregnancy loss, ectopic pregnancy, ongoing pregnancy/delivery fields where available, plurality-related fields, and selected neonatal parameters when documented.

This organization is intentionally aligned with how ART is delivered and discussed in routine care, enabling analyses that can remain close to the clinical sequence of events. Terminology for infertility and ART outcomes was aligned with the International Glossary on Infertility and Fertility Care (7).

### Privacy-preserving design and linkage strategy

A central feature of the OPERA design is its anonymization strategy which, in accordance with GDPR provisions, preserves privacy by being applied prior to data aggregation, preventing the identification of individual patients. Data anonymization, performed according to a protocol approved by the relevant Ethics Committee, was carried out during the extraction phase at each center; identifiers were removed and replaced with a non-identifiable code that does not allow re-identification of the patient or the originating center. The data were then securely transferred via an encrypted channel to the Repository located in Italy on a dedicated server owned by MedITEX. Consequently, OPERA does not retain direct patient identifiers in the central database, nor the individual center codes, and multiple ovarian stimulation cycles cannot be linked to the same woman—each stimulation cycle is treated as an independent unit at the patient level (8).

At the same time, OPERA preserves the ability to link FET cycles to the originating stimulation cycle, enabling analyses consistent with clinically meaningful “stimulation episode” endpoints, including cumulative outcomes across fresh and frozen transfers derived from the same stimulation. This design reflects a deliberate trade-off: stronger privacy protection at the patient level while retaining linkage where it most directly supports clinically interpretable cumulative ART endpoints.

### Data cleaning, harmonization, and quality checks

Because routine documentation can vary across centers and over time, OPERA incorporates structured data cleaning and harmonization steps. In the dataset summarized here, duplicate records were removed (*n* = 1,347). FET cycles that could not be linked to an originating stimulation cycle were excluded (*n* = 43). Cycles with incomplete data due to loss at follow-up were excluded (*n* = 8,524). After these steps, the cleaned dataset included 64,081 OS cycles and 31,027 intended FET cycles.

Harmonization addressed modest platform customizations and center-specific data entry conventions (e.g., numeric separators, measurement units, and language-dependent value labels). Implausible values were reviewed and, when appropriate, recoded as missing. OPERA adopts a pragmatic data-quality framework emphasizing ongoing review of key dimensions such as completeness, uniqueness, validity, and internal consistency, supporting the database as a sustainable research asset rather than a one-time extraction (9).

**Figure 1:**
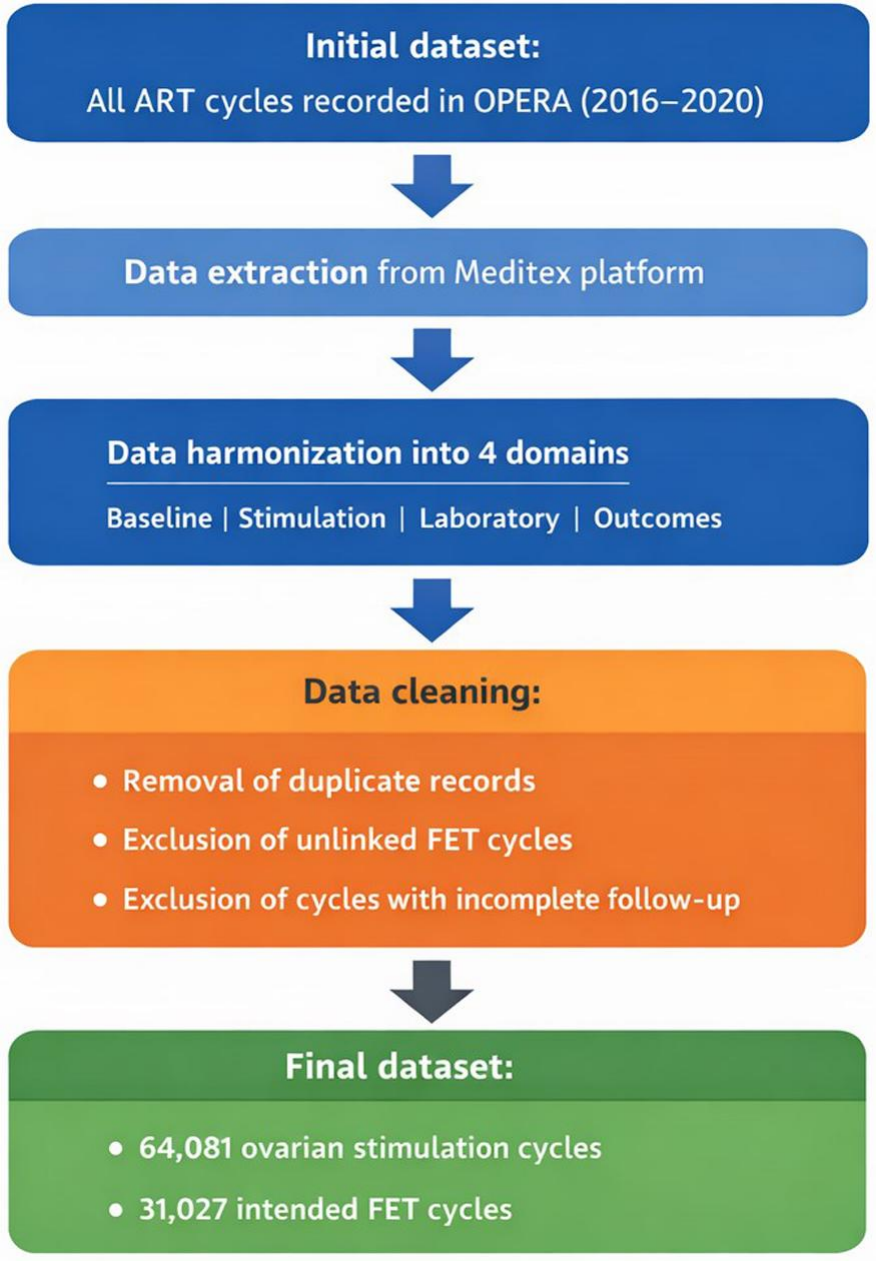
Study flowchart of the OPERA database. Flowchart summarizing data extraction from the Meditex platform, harmonization into four relational domains, and data cleaning procedures, including removal of duplicate records, exclusion of unlinked FET cycles, and cycles with incomplete follow-up. The final dataset included 64,081 OS cycles and 31,027 intended FET cycles.

A flowchart summarizing study design, data extraction, and data cleaning procedures is provided in Figure 1.

### Governance and scientific oversight

OPERA is governed by a Scientific Committee (SC) responsible for oversight of project priorities and database management and for reviewing proposals for studies using OPERA data. The SC reviews internal and external research proposals, assesses feasibility, and approves or rejects studies based on alignment with OPERA objectives, scientific merit, methodological quality, and feasibility within the available data. The SC also oversees publication outputs, manages conflicts or disputes related to data use, and supports risk management strategies relevant to data integrity and privacy.

### Sample size and statistical analysis

No *a priori* sample size calculation was performed, as this study is based on a retrospective real-world database including all eligible cycles recorded during the study period. Continuous variables are summarized using medians with interquartile ranges and means with standard deviations where available. Categorical variables are presented as counts and percentages, with denominators reflecting variable availability (i.e., acknowledging missingness). Given the real-world nature of the dataset, data completeness varied across variables. No imputation was performed. All descriptive analyses were conducted using available-case denominators. No sensitivity analyses were performed, as the aim of the study was descriptive. No hypothesis testing was performed for the descriptive snapshot.

## Results

**Figure 2:**
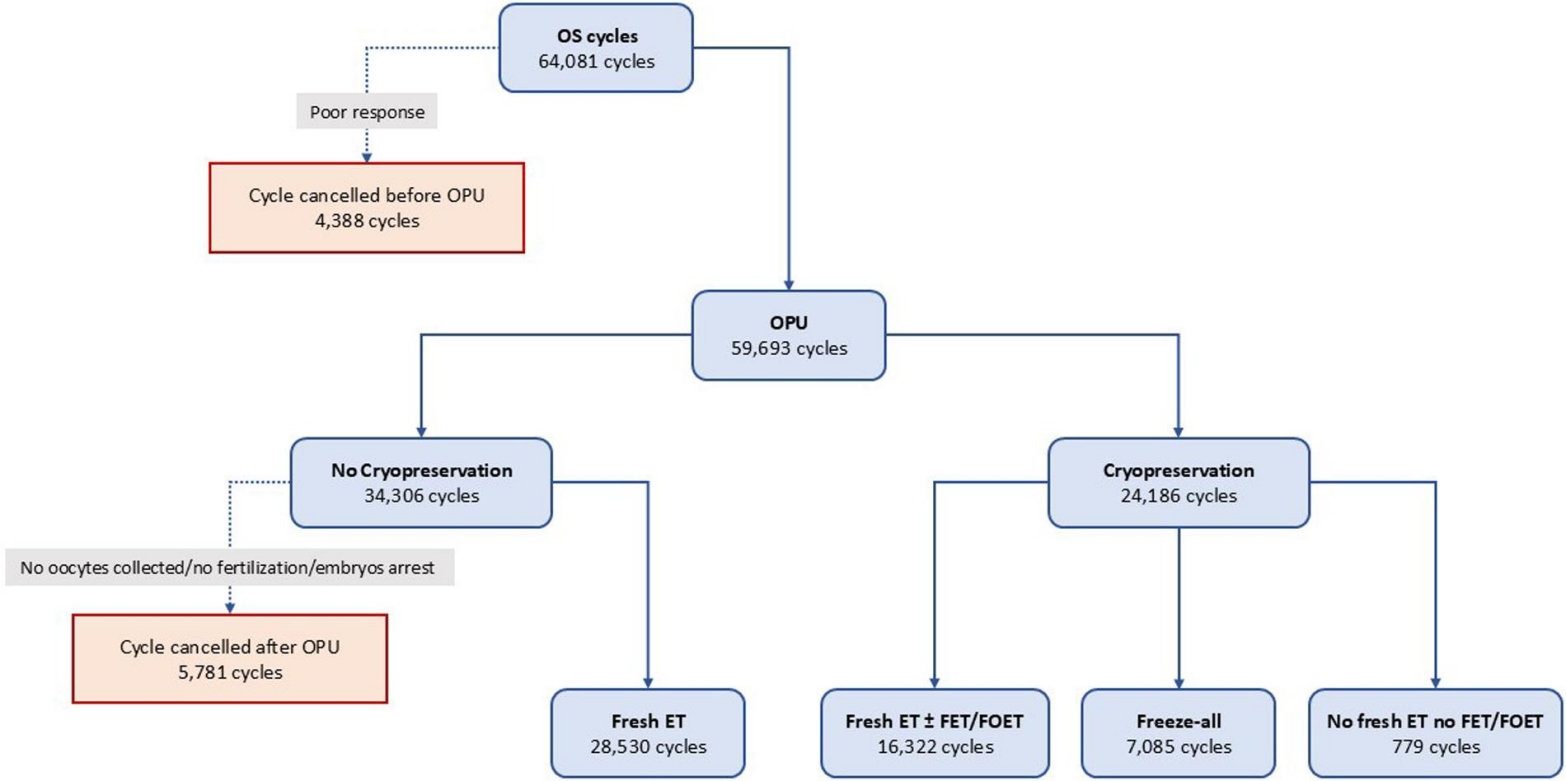
Flow of ovarian stimulation cycles through oocyte retrieval and embryo transfer pathways in the OPERA database (2016–2020). Of 64,081 ovarian stimulation (OS) cycles, 4,388 were cancelled before oocyte pick-up (OPU) because of poor response. OPU was performed in 59,693 cycles. After OPU, 34,306 cycles had no cryopreservation and either ended after OPU (5,781 cycles; no oocytes collected, no fertilization, or embryo arrest) or proceeded to fresh embryo transfer (fresh ET; 28,530 cycles). In 24,186 cycles with cryopreservation, pathways included fresh ET followed by at least one frozen transfer (FET/FOET; 16,322 cycles), a freeze-all strategy (7,085 cycles), or no fresh ET and no subsequent frozen transfer (779 cycles). OS, ovarian stimulation; OPU, oocyte pick-up; ET, embryo transfer; FET, frozen embryo transfer; FOET, frozen oocyte/embryo transfer.

The study workflow, including cycle selection and data cleaning, is illustrated in Figure 2.

After data cleaning and exclusions, OPERA included 64,081 ovarian stimulation cycles and 31,027 intended FET cycles recorded between 2016 and 2020 across 18 centers in Italy and Germany. OPERA's relational architecture maps baseline characteristics, stimulation and triggering, laboratory activity, and pregnancy outcomes, and supports linkage between FET and the originating stimulation cycle.

Female age at OS had a median of 37 years, with male age median 39 years. Median BMI was 22.7 kg/m². Biomarker completeness varied across variables and is reported in detail in Table 1 and subsequent Tables 2–4, where the number and proportion of available observations are provided for each variable. While core demographic variables showed high completeness, some biomarkers such as AFC and basal E2 were available in a smaller proportion of cycles, whereas AMH was available in approximately 61% of OS cycles (median 1.9 ng/mL). Data completeness also varied across other domains, with high availability for treatment-related variables (e.g., gonadotropin exposure and triggering strategy) and lower completeness for selected baseline or hormonal parameters, reflecting differences in data availability across centers.

**Table 1:** Baseline couple characteristics for started ovarian stimulation (OS) cycles, overall and by gonadotropin regimen.

Variable	*N* ^a^	*N* = 64,081
Age of woman at OS	64,080 (100%)	
Median (Q1–Q3)		37 (33–39)
N° of cycles with missing or not applicable data		1
Age of man at OS	63,947 (100%)	
Median (Q1–Q3)		39 (35–43)
N° of cycles with missing or not applicable data		134
BMI	62,375 (97%)	
Median (Q1–Q3)		22.7 (20.6–25.6)
N° of cycles with missing or not applicable data		1,706
Duration of sterility (years)	51,121 (80%)	
Median (Q1–Q3)		4 (3–6)
N° of cycles with missing or not applicable data		12,960
N of previous cycles	64,081 (100%)	
0		24,922 (38.9%)
1		11,186 (17.5%)
2		7,785 (12.1%)
≥3		20,188 (31.5%)
AMH (ng/ml)	39,061 (61%)	
Median (Q1–Q3)		1.9 (0.9–3.5)
N° of cycles with missing or not applicable data		25,020
AFC	1,184 (1.8%)	
Median (Q1–Q3)		9.0 (5.0–13.0)
N° of cycles with missing or not applicable data		62,897
Basal LH (mUI/ml)	38,482 (60%)	
Median (Q1–Q3)		3 (2–5)
N° of cycles with missing or not applicable data		25,599
Basal E2 (pg/mL)	16,690 (26%)	
Median (Q1–Q3)		22 (5–41)
N° of cycles with missing or not applicable data		47,391
Basal P4 (ng/mL)	46,512 (73%)	
Median (Q1–Q3)		0 (0–1)
N° of cycles with missing or not applicable data		17,569
Female factor	60,817 (95%)	
No female factor		20,892 (34.4%)
Endometriosis		5,963 (9.8%)
Ovulatory factor		9,716 (16.0%)
Tubal factor		6,517 (10.7%)
Uterine factor		1,387 (2.3%)
Other factor		16,342 (26.9%)
N° of cycles with missing or not applicable data		3,264
Male factor	51,482 (80%)	
No male factor		13,389 (26.0%)
OAT syndrome		27,761 (53.9%)
Azoo/Cryptozoospermia		3,617 (7.0%)
Unknown nature		1,527 (3.0%)
Other		5,188 (10.1%)
N° of cycles with missing or not applicable data		12,599
Source of sperm (Or intended)	64,080 (100%)	
Fresh		59,816 (93.3%)
Frozen		4,264 (6.7%)
N° of cycles with missing or not applicable data		1

a *N* means the number of cycles for which information is available.

**Table 2:** Patient characteristics by protocol at stimulation start.

Variable	*N* ^a^	*N* = 64,081
GN used	61,490 (96%)	
uFSH		291 (0.5%)
rFSH*α*		20,519 (33.4%)
HMG		14,781 (24.0%)
LH		1,909 (3.1%)
rFSHa/rLH 2:1		14,264 (23.2%)
rFSH*β*		3,891 (6.3%)
Multiple gn used (not Long acting FSH)		3,128 (5.1%)
Long acting FSH (alone or in combination)		1,803 (2.9%)
rFSH*δ*		904 (1.5%)
N° of cycles with missing or not applicable data		2,591
GnRH analog	60,135 (94%)	
GnRH antagonist		47,116 (78.4%)
GnRH agonist		13,019 (21.6%)
N° of cycles with missing or not applicable data		3,946
E2 peak (pg/mL)	52,495 (82%)	
Median (Q1–Q3)		1,028 (358–1,809)
N° of cycles with missing or not applicable data		11,586
P4 peak (ng/mL)	43,246 (67%)	
Median (Q1–Q3)		1 (0–1)
N° of cycles with missing or not applicable data		20,835
Final maturation oocyte triggering	56,650 (88%)	
HCG		44,203 (78.0%)
GnRH agonist		11,146 (19.7%)
Dual triggering		1,301 (2.3%)
N° of cycles with missing or not applicable data		7,431
LPS with progesterone (or intended)	40,398 (63%)	
Vaginal		19,998 (49.5%)
Oral		9,599 (23.8%)
Injection		2,767 (6.8%)
Mixed		7,399 (18.3%)
P4+ GnRH analog		635 (1.6%)
N° of cycles with missing or not applicable data		23,683
OHSS/Risk of OHSS	64,081 (100%)	3,215 (5.0%)
OHSS requiring hospitalization	64,081 (100%)	201 (0.3%)
Cycle cancellation before OPU	64,081 (100%)	4,384 (6.8%)

a *N* means the number of cycles for which information is available.

**Table 3:** Gonadotropin regimens and early laboratory or procedural features.

Variable	*N* ^a^	*N* = 58,461
N of oocytes retrieved	58,461 (100%)	
Median (Q1–Q3)		8 (4–12)
N of M2 oocytes (ICSI—only cycles with complete data)	37,225 (64%)	
Median (Q1–Q3)		6 (3–10)
Method of insemination	57,307 (98%)	
ICSI		41,344 (72.1%)
IVF		13,881 (24.2%)
Both		2,082 (3.6%)
N of oocytes injected/inseminated	58,461 (100%)	
Median (Q1–Q3)		6 (3–10)
N of oocytes fertilized	58,461 (100%)	
Median (Q1–Q3)		6 (3–9)
Cryopreservation	58,461 (100%)	
Yes		24,166 (41.3%)
No		34,295 (58.7%)
N embryos/oocytes/2pn cryopreserved	58,460 (100%)	
Median (Q1–Q3)		0 (0–2)

a *N* means the number of cycles for which information is available.

**Table 4:** Outcomes for IVF/ICSI cycles undergoing to embryo-transfer.

Variable	*N* ^a^	*N* = 44,858
Clinical pregnancy	44,858 (100%)	15,163 (33.8%)
Successful birth (at least 1 newborn) for all fresh ET	44,858 (100%)	11,561 (25.8%)
N of deliveries per cycle	11,518 (26%)	
1		9,695 (84.2%)
2		1,783 (15.5%)
3		40 (0.3%)
N° of cycles with missing or not applicable data		33,340

a *N* means the number of cycles for which information is available.

Gonadotropin exposure was documented in 96% of OS cycles. The most frequently reported categories were recombinant FSH alfa (33.4%), hMG (24.0%), and rFSH alfa/rLH 2:1 (23.2%). Among cycles with recorded GnRH analogue type, GnRH antagonists predominated (78.4%). Triggering strategy was mainly hCG (78.0%), with GnRH agonist trigger (19.7%) and dual trigger (2.3%). OHSS/risk of OHSS was recorded in 5.0%, and OHSS requiring hospitalization in 0.3%; cancellation before oocyte retrieval occurred in 6.8%.

ICSI was the most frequent insemination method (72.1%), followed by IVF (24.2%) and mixed IVF/ICSI approaches (3.6%). Among cycles reaching retrieval, the median number of oocytes retrieved was 8 (IQR 4–12).

Among 44,858 fresh ET cycles, clinical pregnancy was 33.8% and successful birth 25.8%.

Among 29,939 performed FET cycles, clinical pregnancy was 34.2% and successful birth 24.3%.

## Discussion

OPERA was built with a straightforward premise: high-quality ART data already exist in routine clinical care, but they often remain fragmented, locally siloed, and difficult to use for multicenter research at scale. OPERA therefore focused on two priorities from the outset—harmonization and governance—and operationalized these priorities through a shared electronic documentation platform, a standardized extraction pipeline, and Scientific Committee oversight for study feasibility, analysis, and publication processes (4).

The descriptive snapshot of OPERA provides several clinically intuitive signals. First, the median female age of 37 years reflects an ART population that includes a substantial proportion of women of advanced reproductive age, consistent with trends in many European settings. Second, antagonist protocols represented the dominant approach among cycles with documented GnRH analogue type, echoing the widespread uptake of antagonist regimens during the 2010s, driven by patient convenience and OHSS risk mitigation strategies. Third, the predominance of ICSI in routine practice (over 70% of cycles) highlights how insemination strategies in real-world care may diverge from narrow indications and reflect both center policy and perceived efficiency, even as debate continues regarding indications and value in non-male factor infertility (3, 10).

Finally, OPERA demonstrates the ability to capture outcomes across both fresh and frozen transfer pathways. Clinical pregnancy rates were similar between fresh and FET cycles in the snapshot, and successful birth rates were of comparable magnitude. These patterns are consistent with the broader clinical reality that ART success is increasingly shaped by the distribution of embryos across fresh and subsequent frozen transfers, and by strategies such as elective freeze-all, blastocyst culture, and single embryo transfer policies.

Although cycle characteristics are reported, no inferential analyses were performed to evaluate their association with outcomes, as this falls beyond the predefined descriptive scope of the present study. The present report is purely descriptive and does not aim to assess effectiveness or causal relationships. Such analyses require appropriately designed analytical studies and are planned as future work within the OPERA project.

Registries remain essential for surveillance, transparency, and public accountability (3). OPERA was not designed to replace these systems. Instead, OPERA's value lies in its detail and relational linkage, allowing researchers to study questions that are often difficult to address with aggregate reporting. Examples include: comparative effectiveness of stimulation and gonadotropin strategies; associations between laboratory variables and outcomes; evaluation of trigger approaches and OHSS-related endpoints; and analyses of cumulative outcomes derived from a stimulation episode and its derived transfers.

OPERA also places governance at the center of data use. By requiring proposal review, feasibility assessment, and analytic oversight, OPERA aims to ensure that observational analyses are conducted with appropriate methodological rigor and with transparent acknowledgment of limitations inherent to routine datasets.

### Strengths

Key strengths of OPERA include its large scale, multinational scope, and multicenter nature; the repeatability of its extraction pipeline; its relational structure that reflects the real clinical pathway; and explicit governance procedures to support responsible and reproducible research. In addition, OPERA's linkage of FET cycles to the originating stimulation cycle enables clinically meaningful cumulative approaches while preserving privacy.

### Limitations and interpretation considerations

OPERA also shares limitations that are typical of real-world datasets. A key limitation of the OPERA dataset is the variable completeness across clinical variables. As a real-world database derived from routine clinical practice, the completeness of variables within OPERA is inherently variable. Core variables considered essential for clinical documentation are consistently recorded across centers, whereas other parameters depend on local workflows and individual clinician practices and may therefore be inconsistently captured. This reflects the pragmatic nature of real-world data collection and is consistent with similar multicenter observational initiatives. For this reason, the completeness of each variable is explicitly reported. Importantly, missingness should not necessarily be assumed to be random, as it may reflect differences in center-level workflows, documentation practices, and clinician preferences. Consequently, analyses based on available-case denominators should be interpreted with caution. This heterogeneity may introduce bias in analyses restricted to complete cases and should be carefully considered when designing downstream studies.

In addition, outcome capture beyond early pregnancy may be uneven: delivery and neonatal information can be incomplete or inconsistently documented, particularly when follow-up occurs outside the IVF center or when births are recorded in separate healthcare settings. Finally, OPERA is not a population-based registry; it reflects the practice patterns of participating clinics and is not intended to provide national incidence estimates or to be fully representative of all ART activity in Italy and Germany (6, 9). However, the inclusion of multiple centers across two countries and the use of a shared electronic platform support consistency in data capture and provide a meaningful representation of real-world clinical practice within the participating network.

These limitations do not undermine OPERA's purpose. Rather, they represent the practical trade-offs inherent to privacy-preserving, multicenter real-world data initiatives. Importantly, OPERA makes these trade-offs explicit, and decisions about study feasibility, analytical choices, and interpretation are placed under Scientific Committee oversight.

A key interpretative point is that the data presented here represent a descriptive snapshot of clinical practice within a defined historical window (2016–2020). ART practice is inherently dynamic and continues to evolve, particularly in relation to ovarian stimulation protocols, gonadotropin selection, trigger strategies, and freeze-all policies. Since the end of the observation period, additional shifts in protocol preferences and laboratory approaches have occurred in many centers. Accordingly, the present analysis should be interpreted as a time-specific representation of practice patterns rather than a definitive account of current ART care.

OPERA is intended as a living research infrastructure. Future work can extend the database in several directions: expansion of participating centers and years; development of harmonized definitions for additional clinically relevant variables; methodological work addressing missing data and center-level heterogeneity; and development of study templates for comparative effectiveness questions where observational bias is a key concern. Where feasible within regulatory and privacy constraints, linkage to additional data sources (e.g., perinatal outcomes registries or patient-reported outcomes) could further strengthen long-term outcome capture and patient-centered evaluation. This limitation prevents patient-level cumulative analyses across multiple independent stimulation cycles and does not allow the reconstruction of longitudinal patient trajectories across repeated treatments. As a result, OPERA is primarily suited to cycle-level analyses rather than patient-level longitudinal analyses. However, linkage between frozen embryo transfer cycles and the originating stimulation cycle is preserved, enabling cumulative outcome assessment within a single stimulation episode.

OPERA is a harmonized, multicenter real-world ART database spanning Italy and Germany, created from routine electronic records with a standardized extraction pipeline, privacy-preserving anonymization, and Scientific Committee governance. The descriptive snapshot demonstrates its capacity to characterize contemporary ART practice and outcomes and supports OPERA as an enabling platform for reproducible real-world research in reproductive medicine.

## Data Availability

The raw data supporting the conclusions of this article will be made available by the authors, without undue reservation.

## OPERA working group

### Italian OPERA centers

- Prof. Francesco Fusi (ASST Papa Giovanni XXIII, Bergamo)
- Dr. Edgardo Somigliana and Dr. Marco Reschini (Fondazione IRCCS Ca' Granda Ospedale Maggiore Policlinico, Milano)
- Dr. Antonino Guglielmino (UMR HERA, Sant'Agata Li Battiati)
- Dr.ssa Carolina Becattini (Futura Diagnostica Medica PMA, Firenze)
- Dr.ssa Luciana De Lauretis (Istituto Clinico Città Studi, Milano)
- Dr. Stefano Mosca & Dr.ssa Alessandra Fraioli (Ospedale Beauregard, Aosta)
- Dr.ssa Sara Scandroglio (ASST Sette Laghi, Varese)
- Dr.ssa Patrizia Sulpizio (ASST Santi Paolo e Carlo, Milano)
- Dr.ssa Valeria Polli & Dr. Simone Palini (Ospedale Cervesi, AUSL Romagna, Cattolica)

### German OPERA centers

- Prof. Georg Griesinger (Universitäres Kinderwunschzentrum Lübeck und Manhagen)
- Dr. Daniel Noss (Reproduktionsmedizin München – MVZ)
- Dr. Ulrich Göhring (KinderwunschPraxis Dres. Göhring, Tübingen)
- Dr. Andreas Tandler-Schneider (Fertility Center Berlin GbR)
- Dr. Robert Fischer (Fertility Center Hamburg GmbH)
- Prof. Jan Krüssel (UniKiD, Düsseldorf)
- Prof. Konstantin Manolopoulos (MVZ Kinderwunsch- und Endometriosezentrum, Offenbach; European University Cyprus-EUC-Frankfurt)
- Prof. Stefan Kissler (Kinderwunsch- & Hormonzentrum, Frankfurt)
- Dr. Jens Peter Reiher (Kinderwunschzentrum Leipzig-Chemnitz)

Finally, we gratefully acknowledge the essential technical and infrastructural support provided by Advice Pharma, Itamedical, and Critex.
